# Surgery

**Published:** 1888-03

**Authors:** 


SURGERY.—The *Annals of Surgery*, the only English journal published devoted exclusively to surgery, enters now upon its fourth year. Drs. L. S. Pilcher, of Brooklyn, N. Y., and C. B. Keetley, of London, Eng., are the chief editors, assisted by most all the able surgeons of this country as well as Europe, which is sufficient guarantee of the literary merits. We bespeak for it the co-operation of the members of the profession who are interested in progressive surgery. J. H. Chambers & Co., St. Louis, Mo., are the publishers, and deserve great credit for undertaking to produce such an important journal as *Annals*, and for its artistic execution.

---

THE annual meeting of the New York State Medical Society, held at Albany, February 7th, 8th and 9th, was largely attended. Many of the papers presented were of exceptional interest, and several of them will appear in later numbers of the JOURNAL. The following were elected officers for the ensuing year: President—Dr. S. B. Ward, of Albany; Vice-President—Dr. A. W. Suiter, of Herkimer; Treasurer—Dr. C. H. Porter, of Albany; Secretary—Dr. Wm. Manlius Smith, of Syracuse.

